# Inexpensive, scalable camera system for tracking rats in large spaces

**DOI:** 10.1152/jn.00215.2018

**Published:** 2018-07-25

**Authors:** Rajat Saxena, Warsha Barde, Sachin S. Deshmukh

**Affiliations:** Centre for Neuroscience, Indian Institute of Science, Bangalore

**Keywords:** hippocampus, large space, medial entorhinal cortex, spatial navigation

## Abstract

Most studies of neural correlates of spatial navigation are restricted to small arenas (≤1 m^2^) because of the limits imposed by the recording cables. New wireless recording systems have a larger recording range. However, these neuronal recording systems lack the ability to track animals in large area, constraining the size of the arena. We developed and benchmarked an open-source, scalable multicamera tracking system based on low-cost hardware. This “Picamera system” was used in combination with a wireless recording system for characterizing neural correlates of space in environments of sizes up to 16.5 m^2^. The Picamera system showed substantially better temporal accuracy than a popular commercial system. An explicit comparison of one camera from the Picamera system with a camera from the commercial system showed improved accuracy in estimating spatial firing characteristics and head direction tuning of neurons. This improved temporal accuracy is crucial for accurately aligning videos from multiple cameras in large spaces and characterizing spatially modulated cells in a large environment.

**NEW & NOTEWORTHY** Studies of neural correlates of space are limited to biologically unrealistically small spaces by neural recording and position tracking hardware. We developed a camera system capable of tracking animals in large spaces at a high temporal accuracy. Together with the new wireless recording systems, this system facilitates the study of neural correlates of space at biologically relevant scale. This increased temporal accuracy of tracking also improves the estimates of spatiotemporal correlates of neural activity.

## INTRODUCTION

Spatial navigation is a widely employed behavior to study the neuronal circuits underlying cognition, learning, and memory. Since the discovery of place cells in the hippocampus four decades ago (O’Keefe and Dostrovsky 1971; O’Keefe and Nadel 1978), a considerable amount of work has been undertaken to understand the representation of space in the brain. Over the years, a variety of cell types such as grid cells (Hafting et al. 2005), head direction cells (Ranck 1984; Taube et al. 1990), speed cells (Kropff et al. 2015), border cells (Savelli et al. 2008; Solstad et al. 2008), object cells (Deshmukh and Knierim 2011), and landmark vector cells (Deshmukh and Knierim 2013) have been recorded from the hippocampal formation, advancing our understanding of its role in spatial navigation.

Because of the limits imposed by the cables extending from the animal to the recording system, these experiments studying spatial maps have largely been limited to small spaces (≤1 m^2^), with rare exceptions (for example, 1.5 m × 1.4 m in Fenton et al. 2008; 1.8 m × 1.4 m in Park et al. 2011; 2.2 m × 2.2 m in Stensola et al. 2012; 3.5-m-diameter circular arena in Gothard et al. 1996; 18-m track in Kjelstrup et al. 2008; 48-m track in Rich et al. 2014). This experimental constraint leaves a significant lacuna in our understanding of the neural correlates of spatial navigation in environments of scale and complexity comparable to the natural habitat of the rat. Home ranges of Norway rats vary from tens of square meters in urban areas to hundreds of square meters in farms and fields (Lambert et al. 2008; Oyedele et al. 2015); rat burrows occupy an area of the order of 10 m^2^ (Calhoun 1963).

The advent of wireless recording systems enables us to now record neural activity from the hippocampal formation while the rat forages in larger and more complex environments. Most commercial neuronal recording systems, however, do not have provisions for recording from more than two cameras, which again constrains the size of the behavioral arena, leading to an increased need for a system capable of tracking animals in larger environments. Using wide-angle lenses with the standard cameras (with a typical resolution of 640 × 480 or 720 × 560 pixels) supplied with electrophysiology systems is not a satisfactory solution, because the resolution (cm/pixel) decreases drastically with increased area of the field of view. Wide-angle lenses with higher resolution 4K cameras (cameras with horizontal resolution of the order of 4,000 pixels; typical resolution: 3,840 × 1,600 pixels) can alleviate the resolution issue. However, this system is susceptible to occlusion caused by an experimenter or an environmental feature blocking the camera’s line of sight, similar to any other single-camera tracking system. Typical 4K cameras do not have a provision to log time stamps for each frame and thus need to use a temporal synchronization signal (LED flash) to align with the neuronal recording system. Accuracy of the LED synchronization method is limited by the frame rate. Together with the high costs of the 4K camera, occlusion and synchronization issues make this solution suboptimal.

In this article, we describe a novel tracking system comprising eight overhead Raspberry Pi cameras (referred to as the “Picamera system” henceforth), capable of tracking an animal’s position in a large environment. To benchmark the Picamera system, we compared its performance with that of a commercial video tracking system sold as a part of a wireless electrophysiology system. We recorded different cell types from the hippocampal formation using both these video trackers coupled with the wireless electrophysiology system. We show that the higher temporal accuracy of the Picamera system improved our ability to estimate multiple spatial firing characteristics of spatially modulated cells in standard environments used in spatial navigation studies. We then went on to record from a 5.5-m × 3-m arena using the Picamera system coupled with the wireless electrophysiology system to demonstrate our ability to characterize neural correlates of spatial navigation in a large space.

## MATERIALS AND METHODS

### Animals and Surgical Procedures

Four male Long-Evans rats aged 5–8 mo, weighing 450–600 g, were housed individually on a 12:12-h reversed light-dark cycle and habituated to daily handling over 2 wk before surgery. All experiments were performed according to a protocol approved by the Institutional Animal Ethics Committee of the Indian Institute of Science.

Custom-built hyperdrives having 16 tetrodes plus 2 references were implanted over the right hemisphere under surgical anesthesia (80 mg/kg ketamine + 10 mg/kg xylazine followed by 0.5–2% isoflurane for maintenance). The tetrodes targeted different parts of the hippocampal formation in different rats: area CA1 of the hippocampus (2 rats), medial entorhinal cortex (MEC; 1 rat), and MEC and lateral entorhinal cortex (LEC; 1 rat).

### Training and Experimental Protocol

The rats recovered for 5–7 days after the surgery until their weights stabilized. During subsequent training and recordings, the rats were food deprived and trained to forage for food on a 1-m × 1-m black square platform. The neural recordings were performed in multiple setups: a 5.5-m × 3-m room (henceforth referred to as “the large room”), half of this same room, 2.75 m × 3 m, a circular track (diameter = 1 m), a linear track (length = 1 m), and the square platform used for training. Neural signals were recorded wirelessly in all these setups. For small setups (circular track, linear track, and square platform), a single Picamera subunit and a commercial camera were used for position tracking while the rats foraged for food. For the large-room recordings, eight Picamera subunits covering the entire room with substantial inter-camera overlap were used for position tracking while the rats foraged for food.

### Neural Data Recording Hardware

**Fig. 1.**
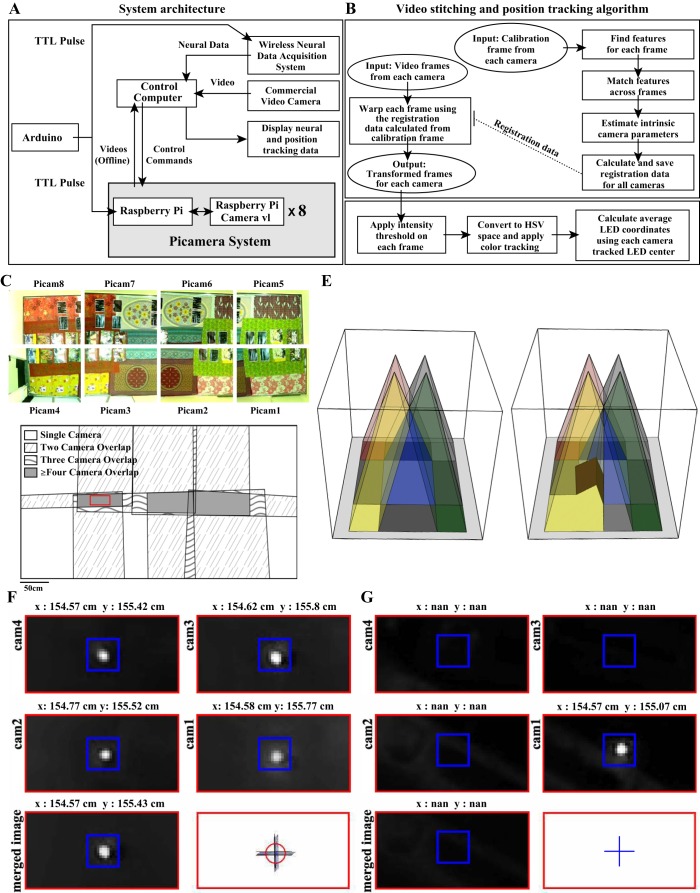
Recording setup. *A*: neural data acquired using the wireless data acquisition system and the video frames monitoring the animal are stored on the control computer. The Neuralynx Cheetah software processes, displays, and stores neural data as well as positions of tracking LEDs on the animal’s head. The Picamera system comprises 8 subunits, each with a Raspberry Pi and a Raspberry Pi camera. Videos recorded from the Picamera are displayed on a monitor and stored locally on each Raspberry Pi. The control computer is used to control all the Picamera subunits and, at the end of the session, receives the video and frame time stamp information acquired by the Picamera subunits for longer term storage. An Arduino microcontroller is programmed to send time synchronization signals [Transistor-transistor logic (TTL) pulses] to all the Picamera subunits and the data acquisition system simultaneously. *B*, *top*: calibration frames from all the cameras are loaded. Scale and rotation invariant features are extracted from each frame and matched across cameras. With the use of geometrically consistent feature matches, intrinsic camera parameters are estimated, followed by calculation of registration data for each camera, which is then saved. Each frame in the video from each camera is then warped and transformed to align it to a single coordinate reference frame using the saved registration data. *Bottom*, intensity thresholding followed by color tracking in HSV (hue, saturation, value) color space is applied on each camera frame to get the coordinates of red and green LEDs. The final position for each frame is estimated after the positions from each camera are averaged. *C*: bedsheets with multiple unique identifiable features were placed in the large room, and an image (calibration frame) was acquired on each Picamera subunit. These features were then used for calculating registration data for each camera. *D*: top-down view of the large room showing the regions covered by individual cameras after registration and alignment of their images for a typical camera setup. Different fills indicate areas of no overlap and overlap between 2 cameras, 3 cameras, and ≥4 cameras. Red rectangular box marks the zoomed-in region of the large room shown in *F* and *G*. *E*: schematic representation (not to scale) of 4-camera overlap region (red rectangle) with no occluding objects (*left*) and with 2 partitions (brown) placed in the environment such that a part of the 4-camera overlap region is visible in only 1 camera (yellow extending into the red rectangle between brown partitions). An LED was placed in the zoomed-in region (marked by red solid line in *D*) in a setup with no occlusion (*F*) and along with 2 boxes (*box 1*, 38 cm × 11.5 cm × 31 cm; *box 2*, 36 cm × 10 cm × 24 cm) occluding the LED such that it was only visible to 1 camera (*G*). *F*, *top*, and *G*, *top*: plots show the zoomed region for transformed images from each individual camera (*cam1–cam4*). *F*, *bottom left*, and *G*, *bottom left*: images shows the zoomed region for a single merged image generated from all 4 cameras using the OpenCV stitching module. *F*, *bottom right*, and *G*, *bottom right*: images shows the zoomed region with the position of the LED marked for each camera-transformed image (black plus sign), the average of each detected position from transformed images (blue plus sign), and that tracked using OpenCV stitching (red circle). The absence of a red circle indicates the intensity fell below the detection threshold. nan, Not a number.

Recordings were performed using the Cheetah data acquisition system and Cube-64, a 64-channel wireless transmitter (Neuralynx, Bozeman, MT). A control computer ran the data acquisition software and stored the acquired data (Fig. 1*A*).

### Video Cameras

The tracking system comprised eight overhead Picamera subunits (Fig. 1*A*). Each subunit had a Raspberry Pi camera module v1 (containing an OmniVision OV5647 ColorCMOS QSXGA 5 MP sensor with f/2.9 aperture lens) connected to a Raspberry Pi 2 model B computer (1 GB RAM, 900 MHz quad-core processor and 32 GB class 10 SD card) (https://www.raspberrypi.org). We recorded video on each Picamera subunit at 640 × 480 resolution at 30 Hz with high temporal accuracy and no dropped frames over 4 h of recording. The Picamera system could acquire at higher frame rates with high temporal accuracy, but there were occasional frame drops (0.22% for 50 Hz and 0.31% for 60 Hz), and thus we chose to acquire at 30 Hz. Each camera has a field of view of 2.43 m × 1.82 m when mounted at a height of 2.8 m. The parallel architecture of the Picamera system, with each Raspberry Pi camera recording video on its own Raspberry Pi computer independently of others, makes it easily scalable without compromising temporal accuracy. The Picamera Python library (https://picamera.readthedocs.io/) was used to acquire video data along with time stamps from the cameras. Video recordings from the Picamera were displayed using a composite video output port available on each Raspberry Pi.

The Picamera was compared with the standard camera kit supplied by Neuralynx for benchmarking. The camera kit (henceforth referred as “commercial camera”) comprised a CV-S3200 analog color camera with a 6-mm lens (JAI, San Jose, CA) and an HVR 1850 PCIe frame grabber card (Hauppauge Digital, Hauppauge, NY), which is compatible with the Cheetah software. The camera covered an area of 3 m × 2.3 m when mounted at a height of 2.8 m. The commercial camera recorded videos at 25-Hz frame rate due to the PAL encoding standard followed for India’s 50-Hz frequency of alternating current (AC) power (the NTSC encoding standard, which records videos at 30 Hz and is routinely used in countries with 60-Hz AC power supply, distorted the video in our setup). Because the Picamera system runs on 5-V direct current (DC) power, it is not affected by the line frequency while acquiring at 30-Hz frame rate. To account for the potential confound due to different frame rates, we compared data recorded in one rat by acquiring video at 25 Hz on both the Picamera and the commercial camera.

### Time Synchronization Across Multiple Picamera Subunits and the Data Acquisition System

Each Raspberry Pi was connected via a DGS-1024D Ethernet hub (D-Link, Taipei, Taiwan) to the control computer and remotely operated over the network using PuTTY (https://www.putty.org/) for secure shell (ssh).

To synchronize position tracking data (see below) acquired in these videos with neural recordings, it is critical to know the time at which each frame was recorded. Default video capture protocols do not record time stamps for each frame. The Picamera Python library offers an option to save operating system clock time, but this option is prone to inaccuracy in time stamping introduced by other processes competing for central processing unit resources. To reduce this inaccuracy in frame time stamps, we modified the Picamera library. Because the Raspberry Pi’s graphics processing unit (GPU) runs its own real-time operating system, it allows saving of frame time stamp with increased temporal accuracy (see results). A custom video output encoder was written to save the Raspberry Pi’s system time clock (STC) value acquired by the GPU each time the Raspberry Pi camera sent a “start of frame” interrupt signal to the GPU. This “presentation time stamp” accurately times each frame of the video.

Transistor-transistor logic (TTL) pulses generated using Arduino Uno REV3 microcontroller (https://store.arduino.cc/usa/arduino-uno-rev3) were sent to all eight Picamera subunits as well as the data acquisition system for synchronizing time across video and neural data (Fig. 1*A*). Time stamps for each TTL input on/off transition were logged on all the Raspberry Pi computers and the data acquisition system. The difference between time stamps recorded on each Raspberry Pi computer and the data acquisition system corresponding to the first TTL on transition gives us the instantaneous temporal offset between these devices. This offset was then subtracted from all subsequent frame time stamps of each video to convert their time stamps to the data acquisition system’s temporal reference frame. Our recordings showed virtually no temporal drift (<13 µs) between the Raspberry Pi computers and the data acquisition system for up to 4 h of recording. Regardless of the extent of drift, subsequent TTL on/off transitions are used to correct drift of the Raspberry Pi clocks from the data acquisition system clock.

Three files were generated on each Picamera subunit: a video file in H.264 format and two csv files: one containing the time stamps for all the frames and the other one holding the time stamps corresponding to each TTL input on/off transition.

### Video Stitching

Videos from each camera were processed offline. A representation of the 5.5-m × 3-m room was created by aligning simultaneously captured video frames from all eight Picamera subunits, each of which covered only a part of the large room and had a substantial overlap with at least two cameras. Because we had the exact time stamp for each frame, we could temporally align each frame accurately across cameras. Video stitching involved two steps: *1*) calculation of registration data for each camera and *2*) generation of aligned images for each camera (Fig. 1*B*).

#### Calculating registration data.

We used Brown and Lowe’s (2007) algorithm to stitch frames from multiple cameras into a single representation. Briefly, one calibration frame (Fig. 1*C*) was acquired from each camera once at the start of each experiment by laying down cloth/paper with uniquely patterned background to increase the number of identifiable features in the large room. We extracted feature vectors that are invariant to image scale and rotation and that also are robust to changes in illumination, noise, and changes in viewpoints for calibration frames from all cameras. These features were matched across all cameras to find the nearest neighbor pixels between two or more cameras. The random sample consensus (RANSAC) algorithm (Fischler and Bolles 1981) was then applied to find geometrically consistent feature matches and generate registration data for each camera. The registration data for each camera included information about focal lengths (*f_x_*, *f_y_*), lens displacement along the *x*- and *y*-axes (*c_x_*, *c_y_*), radial and tangential distortion coefficients, rotation, and translation matrices. It is important to note that the registration data need to be recalculated if the cameras are displaced/rotated from their original location.

#### Generating the aligned image for each camera.

Individual video frames from all the cameras were then transformed using the registration data (calculated once at the beginning of experiment using one calibration frame from each camera; Fig. 1*C*) to get them all in the same coordinate system. Figure 1*D* shows the overlapping region across cameras after their alignment. We modified the OpenCV library Stitcher class (https://opencv.org/) to work with video files. The original OpenCV module code generates a single merged image from the set of simultaneously recorded input images. The default multiband blending approach used to render the merged image performs a frequency-dependent weighted average, blending higher frequency bands over shorter ranges while blending low-frequency bands over larger ranges (Brown and Lowe 2007). In such a blended image, the intensity of LEDs used for tracking the rat would get averaged down, possibly below the threshold used in position tracking when an experimenter or other objects occlude the field of view of one camera but not another (Fig. 1, *E–G*). We modified the code to generate transformed frames from the video of each camera rather than generating a single merged output frame. The individual camera frame transformation enabled us to tackle the problem of occlusion by experimenter as well as other objects by allowing us to perform thresholding operations on an unaveraged image, followed by averaging of the positions estimated by individual cameras (Fig. 1*G*; notice the absence of a red circle corresponding to position tracked from the merged output frame, because the intensity fell below the detection threshold). The use of unaveraged images from all cameras allowed us to detect the LED on the unoccluded camera (blue plus sign overlaid on black plus sign, because the mean position is identical to single camera position). When the LEDs were visible on multiple cameras, individual camera frame transformation would perform as well as the single merged output frame. This can be seen as the close proximity of the four single-camera locations marked by black plus signs, their mean location marked by a blue plus sign, and a merged frame location marked by a red circle (Fig. 1*F*).

### Position Tracking

Red and green LEDs mounted on the animal’s head enabled us to track the animal’s position and head direction. For each camera frame, all the pixels crossing a predefined intensity threshold were converted to the HSV (hue, saturation, value) color space to identify red and green pixels. The appropriate pixel clusters were identified using a custom HSV color range for red (H = 0:10, 160:180, S = 100:255, V = 50:255; OpenCV uses an H range of 0 to 180 instead of the standard 0 to 360) and green colors (H = 50:70, S = 50:255, V = 100:255), and the coordinates of the center of the red and green pixel clusters were noted as the animal’s position for that frame. The animal’s final position corresponding to a frame was calculated by averaging the position estimate across each camera frame for both the red and green LEDs (Fig. 1*B*). Head direction of the animal was calculated using the positions of red (anterior) and green (posterior) LEDs.

### Data Analysis

#### Cluster cutting.

Manual spike sorting with a custom software (WinClust; J. J. Knierim, Johns Hopkins University) was employed to segregate spikes of isolated single units. Each unit was assigned an isolation quality score on a scale of 1 (very well isolated) to 5 (poorly isolated) based on the separation of its cluster from other units and the background together with its interspike interval histogram. A cluster with an isolation quality of 1–3 and firing at least 50 spikes in a session was used for the subsequent analyses. Putative interneurons (mean firing rates >10 Hz; Frank et al. 2001) were excluded.

#### Spatial firing rate maps and place fields.

The position data along with the spike counts were segmented into 4-cm × 4-cm spatial bins for the large room and 2-cm × 2-cm spatial bins for other setups. Times during which the rat moved <2 cm/s and spatial bins where the rat spent <0.4 s were excluded from the analysis. The firing rate map of each cell was calculated by dividing the number of spikes fired in each bin by the time spent there. Rate maps smoothed using the adaptive binning algorithm (Skaggs et al. 1996) were used to calculate spatial information score (see below). Gaussian (σ = 1.25 bins)-smoothed rate maps were used to calculate peak firing rate and place field size and for illustrations. Only place fields with peak firing rate greater than the 25% of the peak firing rate for that cell’s rate map were included for the place field analysis. The size of an individual place field was determined as number of contiguous pixels (minimum: 7) with firing rate greater than 15% of the peak firing rate of that field.

#### Head direction.

Head direction tuning curves were calculated after the total number of spikes fired for each head direction bin (5° bin width) was divided by the amount of time the rat spent facing in that angular bin (Taube et al. 1990) and were smoothed with a Gaussian with σ = 1.25 bins.

#### Spatial information.

A spatial information score (Skaggs et al. 1996) was used to quantify the spatial tuning of single units. The score calculates the information (in bits) about the rat’s location conveyed by a single spike. We employed a shuffling procedure to estimate the probability of obtaining the observed spatial information by chance. The spike train was shifted cyclically with respect to position data 1,000 times by adding a uniformly generated random number lying between 30 s and the duration of the recording session minus 30 s. The fraction of time-shifted information scores greater than or equal to the observed information score was used to calculate the probability of obtaining the observed information score by chance. A significance threshold of *P* < 0.01 was used to identify neurons with statistically significant spatial information (Deshmukh and Knierim 2011).

#### Theta phase precession analysis.

Theta peaks in the local field potentials were detected as described by Deshmukh et al. (2010). Each spike was then assigned a phase (between 0° and 360°) using linear interpolation between consecutive peaks (Skaggs et al. 1996). For the circular track, two-dimensional (2D) data were transformed into units of degrees on the track for linearized position estimates. Theta phase at which a place cell fired was plotted as a function of linearized position at which it fired to visualize theta phase precession as the animal passed through the place field.

#### Statistical analysis.

Two-tailed tests were used for all quantitative statistical comparisons. Interframe intervals (IFI) for both camera systems were normally distributed; the two-sample *F*-test for equal variances was used for comparing the two. The Wilcoxon signed rank test was performed for all paired comparisons.

### Code Availability

Hardware setup instructions and video data acquisition codes are available at https://github.com/DeshmukhLab/PicameraPaper.

## RESULTS

### Frames Acquired Using the Picamera System Are Temporally More Stable Compared with a Commercial Camera

**Fig. 2.**
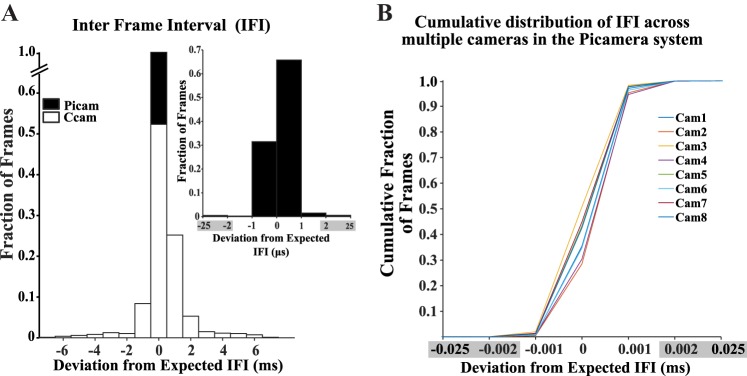
Stability of interframe intervals (IFIs). *A*: jitter in the IFI for a video recorded by the commercial camera (Ccam) and one camera from the Picamera system (Picam). In a 4-h video recording session, the Picamera showed a jitter of less than ±1 ms from the expected IFI vs. ±7 ms jitter shown by the commercial camera. *Inset* shows a zoomed-in plot for IFI for the Picamera showing a jitter of ±0.025 ms. *B*: all 8 Picamera subunits (*cam1–cam8*) had a similar cumulative distribution with most of the IFIs lying within the ±0.002-ms window for a 4-h session. Because there is an extremely low fraction of frames outside ±0.002-ms deviation, in place of the 0.001-ms bin width used for the *inset* histogram in *A* and cumulative distribution plot in *B*, a larger bin width was used to combine data from ±0.002 to ±0.025 ms (marked by gray-shaded regions on the *x*-axis to indicate substantially larger bin width compared with other bins on the same plot). Ccam, commercial camera; Picam, Picamera system.

Performance of the Picamera system was benchmarked against a commercial camera obtained as a part of the Neuralynx Cube-64 wireless recording system. Figure 2*A* shows the fraction of frames showing deviations from the expected IFI for a video recorded for 4 h by the commercial camera and the Picamera in the same session. We defined jitter as the range of deviation from the expected IFI. The Picamera jitter of ±0.025 ms was lower than the ±7-ms jitter of the commercial camera. Thus the Picamera system shows higher temporal accuracy over a long recording session compared with the commercial camera, giving two orders of magnitude improvement in jitter.

Because the data acquisition system uses a frame grabber to record video streaming from a camera, we tested whether webcams show a better temporal accuracy, since some behavior monitoring systems use webcams. We recorded videos using a Logitech C170 USB webcam (Logitech, Lausanne, Switzerland) at 25 Hz. The webcam dropped an average of 8.26% of the frames, giving us an extremely variable IFI (jitter = −28 ms to + 88 ms). Thus webcams may not be ideal for use with neurophysiology systems.

### Reduced Jitter Improves Estimate of Neural Correlates of Behavior

**Fig. 3.**
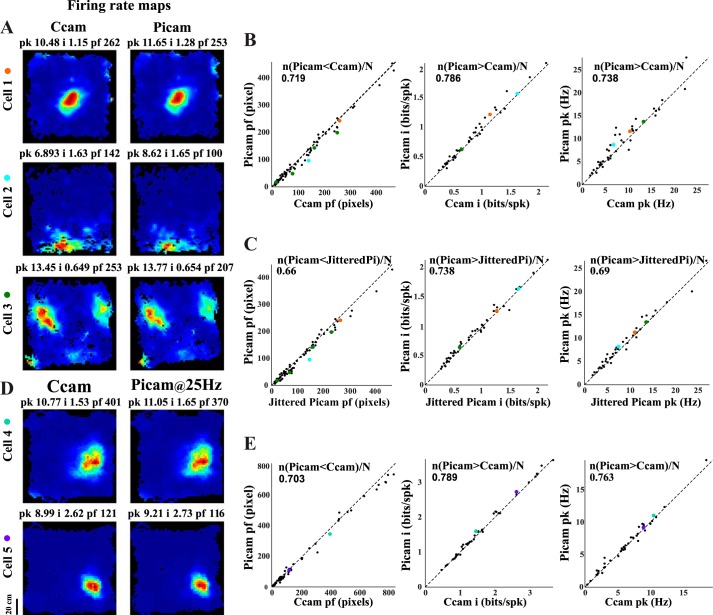
Effect of temporal accuracy of video tracking on estimates of neural correlates of space. *A*: spatial firing rate maps of place cells (*cells 1* and *2*; recorded from dorsal CA1) and a putative grid cell (*cell 3*; recorded from medial entorhinal cortex) computed from videos recorded simultaneously on the commercial camera (Ccam; *left*) and the Picamera (Picam; *right*). Peak firing rate (pk; in Hz), spatial information score (i; in bits/spike), and place field size (pf; in pixels) for each rate map are indicated at *top* of each image. Only the largest field size is presented for an individual cell (although other smaller fields are included in the analysis). For example, grid cell (*cell 3*) field size for the commercial camera is 253 (other field sizes: 165, 80, 17) and that for the Picamera is 207 (other field sizes: 149, 48, 12). *B*: scatter plots for place field size, spatial information, and peak firing rate for both cameras. Identity line (slope = 1) is marked on each plot. All place fields of a single cell meeting criteria in both the cameras were included in the analysis. *C*: scatter plots for place field size, spatial information score, and peak firing rate are calculated for the Picamera and Picamera data with frame times jittered using IFI distribution of the commercial camera (JitteredPi). *D* and *E*: comparison of Picamera videos recorded at 25 Hz with the commercial camera video recorded simultaneously. *D*: spatial firing rate maps of place cells computed from videos recorded at 25 Hz simultaneously on the commercial camera (*left*) and the Picamera (*right*). *E*: scatter plots for place field size, spatial information score, and peak firing rate for the Picamera data recorded at 25 Hz and for the commercial camera. The fraction of data points showing better performance with the Picamera is shown at the *top* of each of the scatter plots in *B*, *C*, and *E*. Each cell shown in *A* and *D* was assigned a color, which was then used to display that cell’s firing characteristics in the scatter plots in *B*, *C*, and *E*.

We tested if the camera jitter affects our assessment of neural correlates of behavior by performing an explicit comparison of the Picamera and the commercial camera recording videos simultaneously with Cube-64 wireless transmitter recording neural activity. Multiple single units were recorded from the hippocampal formation while rats foraged for a food reward in different behavioral arenas (1-m × 1-m platform, 1-m-diameter circular track, 1-m-long linear track) across days. The animal’s position and head direction were estimated from the videos recorded using both the commercial camera and a single Picamera subunit. Spatial firing rate maps for units with significant spatial information score (spatial information >0.25 bits/spike, *P* < 0.01 using rat position estimates from at least 1 of the 2 cameras; *n* = 42) were generated for rat positions estimated from both the cameras used. Figure 3*A* shows firing rate maps for different cell types (2 dorsal CA1 place cells and 1 putative grid cell recorded from MEC) recorded using the commercial camera and the Picamera. The place fields of spatially responsive neurons showed better tuning in the Picamera data than in the commercial camera data. At the population level, place field size was significantly smaller for the Picamera data as opposed to the commercial camera data (Wilcoxon signed rank test, *z* = −3.88, *P* = 0.0001), presumably due to the greater temporal accuracy of the Picamera in positioning the animal (Fig. 3*B*). These results motivated us to look for differences in spatial information and peak firing rate. As expected, peak firing rate (Wilcoxon signed rank test, *z* = 3.14, *P* = 0.0017) and spatial information (Wilcoxon signed rank test, *z* = 3.86, *P* = 0.00011) for the Picamera was significantly greater than that for the commercial camera, after Holm-Bonferroni correction for multiple comparisons (Holm 1979).

We asked whether higher jitter in frame time stamp can lead to a large enough error in assignment of positions to individual spike time stamps to cause degradation of measures of spatial selectivity we compared above. We added jitter to the Picamera IFIs by sampling (with replacement) from the commercial camera IFI distribution, and generated rate maps using the jittered frame time estimate. There were significant reductions in spatial information (Wilcoxon signed rank test, *z* = 3.29, *P* = 0.001) and peak firing rate (Wilcoxon signed rank test, *z* = 2.99, *P* = 0.0028) and an increase in place field size (Wilcoxon signed rank test, *z* = −3.13, *P* = 0.0018) from the Picamera to the jittered Picamera (Fig. 3*C*). These results are consistent with the suggestion that higher temporal accuracy in the Picamera frame time stamps led to more accurate instantaneous position assignment and therefore improved measures of spatial selectivity.

We also tested whether the marginally higher frame rate of the Picamera (30 Hz) compared with that of the commercial camera (25 Hz) can explain the observed improvements by recording 38 single units at 25-Hz frame rate for both the systems in one rat. Consistent with the previous observations, the cells showed better spatial correlates with the Picamera. The Picamera showed smaller place field size (Wilcoxon signed rank test, *z* = −3.1, *P* = 0.002), higher spatial information score (Wilcoxon signed rank test, *z* = 2.87, *P* = 0.0041), and higher peak firing rate (Wilcoxon signed rank test, *z* = 2.53, *P* = 0.011), similar to the results shown earlier (Fig. 3, *D* and *E*). This continued better performance of the Picamera despite a reduction in frame rate indicates that the marginally higher frame rate of the Picamera (30 vs. 25 Hz) in the recordings above may not explain the improvements in the estimation of neural data.

**Fig. 4.**
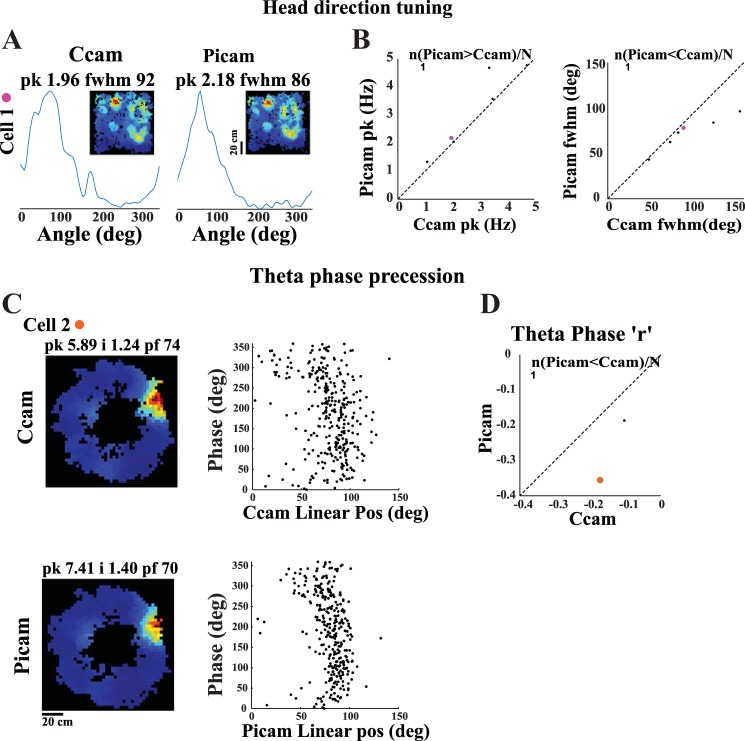
Effect of temporal accuracy of video tracking on head direction tuning and theta phase precession estimates. *A*: head direction tuning plot and spatial firing rate map (*inset*) for a parasubicular head direction-modulated cell computed from videos recorded simultaneously on the commercial camera (Ccam; *left*) and the Picamera (Picam; *right*). Peak firing rate (pk; in Hz) and full width at half maximum (fwhm; in deg) are indicated at the *top* of the head direction plot. *B*: scatter plot for peak firing rate and fwhm for both the cameras. Identity line (slope = 1) is marked on each plot. *C*: spatial firing rate maps (*left*) and theta phase precession plots (*right*) for a hippocampal place cell computed from videos recorded simultaneously on the commercial camera (*top*) and the Picamera (*bottom*). *D*: scatter plot showing Pearson’s correlation coefficient (*r*) for both cameras. The fraction of data points showing better performance with the Picamera is shown at the *top* of each of the scatter plots in *B* and *D*. Each cell shown in *A* and *C* was assigned a color, which was then used to display that cell’s firing characteristics in scatter plots in *B* and *D*. Pos, position; i, spatial information score; pf, place field size.

Next, we tested whether estimates of other neural correlates of behavior also improve with reduced jitter in the new system. Figure 4*A* shows head direction tuning curves of a parasubicular head direction cell recorded using the commercial camera and the Picamera. The head direction tuning appeared sharper for the Picamera than for the commercial camera. For all six head direction-modulated units in our data set, the Picamera showed better estimates of head direction tuning (higher head direction peak firing rate and smaller full width at half maximum) compared with the commercial camera (Fig. 4*B*). The number of units is too small to perform the Wilcoxon signed rank test performed for earlier comparisons, but it is sufficient to perform a test of proportions, testing the null hypothesis that 50% of the neurons will show better head direction tuning with the Picamera. The proportion of neurons showing better performance with the Picamera is significantly higher than that expected by chance (6/6, *Z* = 2.4495, *P* = 0.0071). Figure 4*C* shows theta phase precession (O’Keefe and Recce 1993) of a hippocampal place cell recorded using the commercial camera and the Picamera. Theta phase precession appeared better defined for the Picamera than the commercial camera. To confirm that the apparent improvement in phase precession for each unit was statistically significant, we fit its theta phase precession curve with a straight line. We then calculated the difference between the linear position predicted by this best fit line and the actual position for each spike recorded on each of the video tracking systems (*d*_p_). Variance of *d*_p_ across spikes was used as an indication of tightness of theta phase precession estimate. The Picamera showed tighter theta phase precession estimates for both units compared with the commercial camera (2-sample *F*-test for equal variances: *cell 1*, $\sigma_{\text{commercial camera}}^{2}$ = 524.18, $\sigma_{\text{Picamera}}^{2}$ = 226.57; *f* = 2.31, df_commercial camera_ = 315, df_Picamera_ = 315, *P* = 2.52 × 10^−13^; *cell 2*: $\sigma_{\text{commercial camera}}^{2}$ = 933.78, $\sigma_{\text{Picamera}}^{2}$ = 343.10; *f* = 2.72, df_commercial camera_ = 109, df_Picamera_ = 109, *P* = 3.19 × 10^−7^). Although the number of phase precessing neurons (*n* = 2) is too small for a statistical comparison at the population level (Fig. 4*D*), the anecdotal observations match our expectation of improved phase precession estimate as a consequence of improved temporal accuracy of position estimates.

### Low Jitter in Individual Picamera Frame Timing Allows Precise Synchronization Across Multiple Cameras

**Fig. 5.**
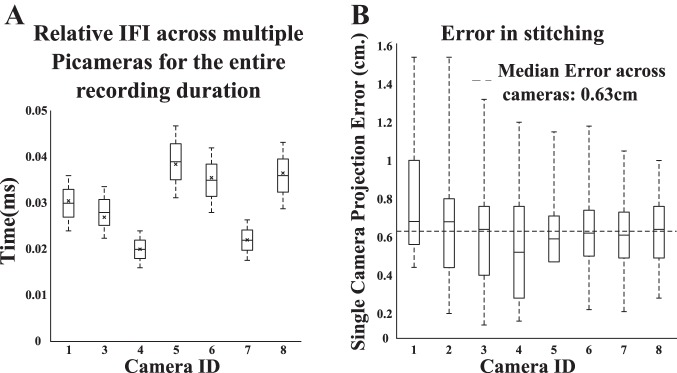
Spatial and temporal accuracy across multiple Picamera units. *A*: relative interframe interval (IFI, or time difference) across cameras for all frames relative to the first camera to start recording the video (*camera 2*). Relative time difference for the reference camera from itself is always 0, and hence *camera 2* is excluded from this plot. The central line, top edge, bottom edge, top bar, and bottom bar of each box represent the 50th, 75th, 25th, 100th, and 0th percentiles, respectively, of the samples. The black cross in each box indicates the start frame time difference across cameras relative to the first camera to start recording the video (*camera 2*). *B*: projection error for each camera, defined as the distance between an intersection point formed after placement of multiple strings to form a grid across the large room for that camera and its corresponding position across all cameras with fields of view overlapping at the intersection point. The central line, top edge, bottom edge, top bar, and bottom bar of each box represents the 50th, 75th, 25th, 100th, and 0th percentiles, respectively, of the samples. Dashed line represents median projection error across cameras. Maximum error noticed was 1.54 cm for a single camera, and median error across cameras was 0.63 cm. ID, identification number.

The commercial camera has ~40% of the frames outside 1-ms deviation from the expected IFI. Using multiple such cameras with their uncorrelated noise in frame time stamps can lead to higher chances of temporal misalignment of frames across cameras. This increased misalignment across cameras could worsen the estimates of neural correlates of behavior even more than in the single-camera case. The low jitter in IFIs of the Picamera subunits discussed earlier predicted that their consecutive frames would be temporally closely aligned with frames from other Picamera subunits, provided all the Picamera subunits started recording videos nearly simultaneously. To test this prediction, we looked at how stable the inter-camera frame interval stayed over the duration of the 4-h recording session across eight Picamera subunits (Fig. 5*A*). In multiple recording sessions, the starting times of the eight cameras are within 0.5 ms of the first camera. Given the low IFI jitter across cameras, in multiple sessions, the consecutive frames from the eight Picamera subunits do not differ from that of the camera with shortest starting lag by more than 0.5 ms. The example in Fig. 5*A* shows an across-camera frame time difference of <0.05 ms for all frames recorded over a 4-h session. The extremely low inter-camera frame timing difference shows that the entire system remained in sync during the recording session, facilitating temporal alignment of video frames across cameras at a submillisecond accuracy.

With the use of a video stitching algorithm, frames from individual Picamera subunits were aligned in a single coordinate system that represented the entire maze (Fig. 1*D*). The aligned frames were then checked for any distortions that could have been introduced because of the stitching algorithm, by using an estimate independent of the calibration frames used for stitching. A grid was formed by stretching multiple strings across the length and breadth of the behavior arena, which gave multiple intersection points between strings running orthogonally for each camera. When an intersection point is visible on multiple cameras, perfect alignment across cameras should place this intersection point at exactly the same *x* and *y* coordinates on the aligned frames of all the cameras. Thus the difference in estimates from different cameras of positions of the shared intersection points provides a measure for accuracy of spatial location alignment using our stitching algorithm. We calculated the projection error for each camera, defined as the distance between an intersection point for that camera and its corresponding position across all cameras with overlapping fields of view. The maximum projection error for single camera with respect to others came out to be 1.54 cm, and median error across cameras was 0.63 cm (Fig. 5*B*). Thus the stitched image has a spatial jitter of less than the pixel size (4 cm) used for creating firing rate maps of neurons when rats foraged in the large room.

### Tracking Spatial Selectivity of Neurons from the Hippocampal Formation in a Large Room

**Fig. 6.**
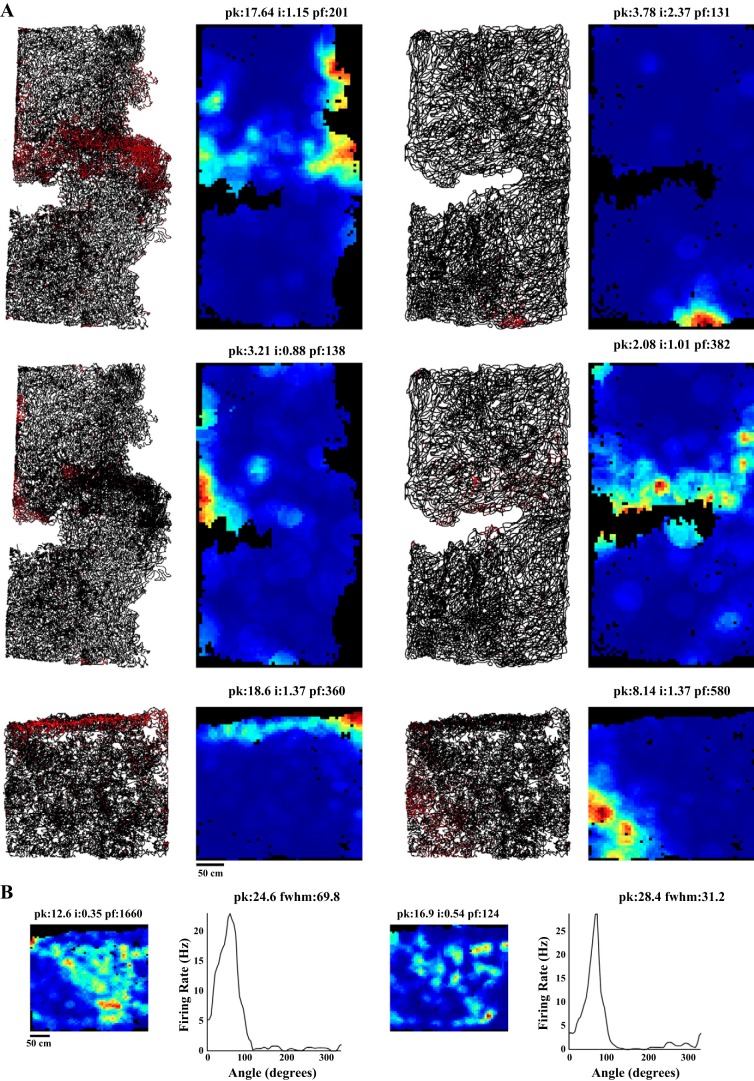
Neural representations of large spaces. *A*: trajectory plots and rate maps for spatially selective cells recorded from ventral dentate gyrus in a 5.5-m × 3-m room (*left*) and a 2.75 m × 3 m room (*right*). Black lines in the trajectory plots mark the trajectory of the rat, whereas red dots indicate locations where the neuron fired. Because of the large number of spikes of the *top left* unit in a region with high occupancy, the trajectory of the rat is not clearly visible in the central band of the trajectory plot despite reduction of the size of the spike dots to 75% of the size used in other plots. The trajectory plot at *middle left* is from a neuron recorded simultaneously, and hence it can be used to see the density of occupancy below the spikes in the central band of the *top left* trajectory plot. *B*: rate maps and head direction tuning curves for 2 head direction cells recorded from parasubiculum in a 2.75-m × 3-m behavior setup. pk, Peak firing rate (in Hz); i, spatial information score (in bits/spike); pf, place field size (in pixels); fwhm, full width at half maximum (in deg).

Multiple Picamera subunits were used to track position of a rat foraging in the large room. Videos recorded from each camera were aligned in a single coordinate system, and the rat’s position was calculated after position was averaged from transformed frames across cameras. Spatial firing rate maps were generated for neurons active during the behavior. Figure 6*A* shows trajectory plots and corresponding spatial firing rate maps for multiple spatially selective cells recorded from the ventral dentate gyrus in a 5.5-m × 3-m room and 2.75-m × 3-m room. These cells showed a range of responses including one (Fig. 6*A*, *top right* and *bottom right*) to a few (*top left*, *middle left*, and *middle right*) place fields and border cell-like activity (*bottom left*). Figure 6*B* shows head direction tuning curves for two head direction cells recorded from the parasubiculum recorded in a 2.75-m × 3-m behavior room. These head direction cell tuning curves in large space look qualitatively similar to the head direction cell tuning curves recorded in standard environments.

**Fig. 7.**
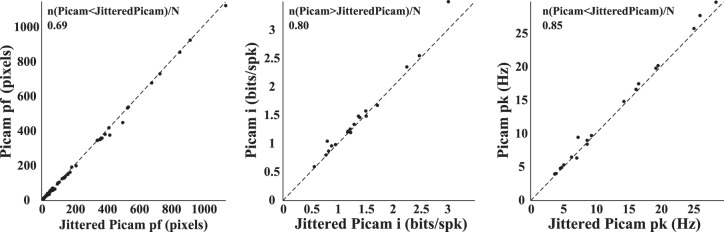
Effect of temporal accuracy of video tracking on estimates of neural representations of large spaces calculated using the 8-camera Picamera system. Scatter plots for place field size (pf), spatial information (i; bits/spike), and peak firing rate (pk) show the Picamera (Picam) and jittered Picamera (jitteredPicam) data for 20 units. Identity line (slope = 1) is marked on each plot. The fraction of data points showing better performance with the Picamera compared with the jittered Picamera is shown at the *top* of each scatter plot.

We hypothesized that the spatial selectivity estimates of neurons recorded in large spaces using multiple cameras would be affected by IFI jitter, but we did not have a commercial camera system with eight cameras capable of tracking rats in large spaces to test this hypothesis. Instead, we employed the strategy used in Fig. 3*C*. We added jitter to the IFIs of each of the eight Picamera subunits for large-room recordings by sampling (with replacement) from the commercial camera IFI distribution (obtained from 72.5 h of recordings performed over 52 days of recording). We compared the spatial selectivity estimates obtained from rate maps generated using these jittered video data with the native video data. There was a significant reduction in spatial information (*n* = 20; Wilcoxon signed rank test, *z* = 2.74, *P* = 0.0062) and peak firing rate (Wilcoxon signed rank test, *z* = 2.94, *P* = 0.0033) and an increase in place field size (Wilcoxon signed rank test, *z* = −4.81, *P* = 1.53 × 10^−6^) from the Picamera to the jittered Picamera (Fig. 7). These results suggest that multiple cameras with higher jitter in frame time stamps can lead to degraded measures of spatial selectivity.

## DISCUSSION

The availability of wireless recording systems now facilitates recording of neural activity in large spaces. However, the commercially available extracellular electrophysiology systems still face limitations in terms of the number of cameras (usually 1or 2) used for tracking rats, which constrains our ability to accurately track them in large environments. In this report, we have demonstrated our ability to record from large spaces by characterizing neural activity in a 5.5-m × 3-m room using the Picamera system with 8 subunits (Fig. 6). The stitching and position estimation algorithms used for tracking the rat in the large room in the current study are completely automated and only require the registration data to be calculated once beforehand. The stitching algorithm gives a maximum error of 1.54 cm in estimating the rat’s position. This error is less than the resolution used for generating spatial firing rate maps. Also, our approach of estimating the rat’s position from registered image from each camera followed by averaging the position estimated from multiple cameras enables us to overcome occlusions in areas with multicamera overlaps. Thus the Picamera system along with the wireless recording system can now be used to perform experiments in larger sized as well as complex environments with occlusions.

Recent studies have tried to address the relationship between environmental scale and spatial representation. A single place cell typically has a single place field in a small environment. Explicit comparisons of hippocampal representations of small (68- and 76-cm-diameter cylinders) and large (1.5 m × 1.4 m and 1.8 m × 1.4 m) 2D environments reveals that the number of place fields per cell increases with an increase in the recording area (Fenton et al. 2008; Park et al. 2011). In contrast, single place field seems to be the norm for place cells recorded on an 18-m-long track (Kjelstrup et al. 2008). On an even longer, 48-m track, hippocampal neurons show a negative binomial distribution of number of place fields per cell such that most cells have 0–2 fields (Rich et al. 2014). The discrepancy between the Fenton et al. (2008) and Park et al. (2011) studies on one hand and the Kjelstrup et al. (2008) and Rich et al. (2014) studies on the other hand can be explained either as the difference between the mechanisms of encoding 1D and 2D environments or as an effect of spatial scale in 1D studies being substantially larger than that in the 2D studies. The second hypothesis predicts that as the spatial scale increases beyond the scales used in the 2D large-space studies, the distribution of the number of place fields per cell will be skewed further with a long tail on the right, rather than shifted rightward. Distinguishing between these two possibilities requires the recording of hippocampal activity from rats foraging in substantially larger 2D spaces such as the 16.5-m^2^ space we recorded from in this study.

A range of methods have been used to track animals in large spaces to study neural representation of space. Each of these methods, including the one we describe here, have their advantages, disadvantages, and limitations. Whereas the large environments used in the Fenton et al. (2008) and Park et al. (2011) studies are small enough to be recorded using single cameras (a stereo camera was used in Park et al. 2011), larger environments create new challenges. Kjelstrup et al. (2008) used a laser beam at one end of the track to sample the rat’s position at 5–20 Hz. In addition to this low and variable temporal resolution, the spatial resolution is also low for this method because it tracks any part of the rat’s body reflecting the laser beam. This simple and elegant design is well suited for tracking rats in large linear spaces, but adapting it to 2D spaces is not trivial. Rich et al. (2014) used three wide-angle cameras to track rats running on a 48-m track winding around a large room. Position of the rat was tracked manually in the videos from the three cameras, followed by manual reconstruction of the track. Although this method works well for a track, it is labor intensive and is likely to run into problems when the rat is tracked in 2/3D. Yartsev and Ulanovsky (2013) recorded 3D positions of freely flying bats by using two cameras placed in two upper corners of the room. Their median position reconstruction accuracy was 1.2 cm, which is very good, especially considering the fact that they were performing 3D reconstructions using only two cameras. Neither Rich et al. (2014) nor Yartsev and Ulanovsky (2013) reported temporal jitter or spatial resolution of the cameras. Tsoar et al. (2011) used GPS data loggers sampling at <0.1 to 1 Hz to track bats flying tens of kilometers in their natural habitat. Although they do not mention the spatial resolution, typical GPS resolution is about a few meters. Whereas video tracking systems obviously cannot compete with this system for the sheer spatial scale required for tracking bats in their natural habitats, the low spatial and temporal resolution of this system will allow only a coarse estimate of spatial correlates of neurons if this GPS system is coupled with electrophysiological recordings.

A thorough comparison of our method with the methods described above (or with other popular commercial hardware combining video tracking with neurophysiology) is impossible in the absence of data such as temporal accuracy, spatial resolution, etc. However, our results should be sufficient to convince the reader of the necessity of calibrating/benchmarking one’s tracking systems, whether they are custom made or purchased off the shelf. Systems capable of keeping track of exact time when each frame was gathered (e.g., the “start of frame” interrupt signal used by the Picamera to indicate start of frame acquisition) will perform well. In contrast, systems with variable data transfer rates (e.g., those using frame grabber cards, USB cables, etc.) will show higher jitter unless the cameras accurately time stamp individual frames before transferring them to the computer.

The Picamera system is temporally more accurate than a commercially available system used for benchmarking in this study (Fig. 2). It is easily scalable (because of its parallel architecture, adding more Raspberry Pi cameras to cover arbitrarily large areas is trivial) and can be adapted for use with complex environments with multiple occlusions. The Picamera system is cost effective because of the use of low-cost, off-the-shelf, easily available components (prices obtained from https://www.element14.com: Raspberry Pi 2 model B + Raspberry Pi camera module v1: $35 + $27 per unit, Arduino: $10) and makes the collection of high-quality, temporally accurate behavioral data sets in large spaces feasible. Open-source libraries used in developing the code, OpenCV (used in stitching and position tracking) and the Picamera Python library (used in acquiring videos and time stamp data), made it possible to customize the code, and, in turn, allowed us to record videos at submillisecond temporal accuracy. Potential users of this system will do well to keep in mind certain requirements and limitations of this system, in addition to the advantages listed above, while considering adoption. Extended recordings using multiple cameras occupy substantial storage space: ~0.5 GB/h per camera at 640 × 480 resolution and 30-Hz frame rate. Storing only the extracted positions from stitched videos substantially reduces the storage space required. Although sufficient for acquiring videos at high temporal accuracy, the slow processor and GPU speeds of the Raspberry Pi preclude online tracking at high temporal accuracy for use with closed-loop systems. The Picamera system uses video stitching techniques to align camera frames. The stitching algorithm requires substantial overlap (typically >30%) between cameras for estimating individual camera parameters accurately. Thus the area covered by this system is substantially less than the sum of areas covered by individual cameras. With a substantial overlap in the fields of view of multiple cameras, this system can be adopted for 3D tracking, but we have not yet implemented 3D tracking algorithms.

Although we designed the Picamera system as a system to track rats in large spaces, it also substantially improves our estimates of a number of neural correlates as a single-camera system. Improvement in spatial and temporal accuracy of a tracking system is expected to lead to a reduction in noise of estimation of behavioral variables such as instantaneous position and head direction. This improved accuracy in tracking behavioral variables should lead to reduction in noise introduced by the tracking system in our estimates of spatial selectivity and head direction tuning of neurons. Predictably, the rate maps generated using the Picamera had significantly smaller place field size and increased peak firing rate and spatial information content compared with a commercial system with higher temporal jitter (Fig. 3). Similarly, the Picamera showed sharper head direction tuning and, anecdotally, tighter theta phase precession compared with the commercial system (Fig. 4). An understanding of mechanisms and functions of theta phase precession will benefit from increased accuracy in quantifying theta phase precession. Whereas linear (O’Keefe and Recce 1993) or circular-linear (Huxter et al. 2008; Kempter et al. 2012) regression is routinely used to quantify theta phase precession, the observed dynamics of theta phase precession are more complex (Mehta et al. 2002; Skaggs et al. 1996; Souza and Tort 2017; Yamaguchi et al. 2002), with the rate of precession changing as a function of location within the place field. Different proposed mechanisms for theta phase precession predict different shape of theta phase precession and variability of the preferred theta phase at different locations within the place field (reviewed in Burgess and O’Keefe 2011). Improved accuracy of the position tracker will clearly enhance our estimate of the shape as well as variability of theta phase precession, enabling us to distinguish between the different models. The Picamera system with its submillisecond temporal accuracy is well suited for such applications, even at the current frame rate of 30 Hz, but this can further improve at higher frame rates. Although the current system is capable of acquiring at 60 Hz with minimal (0.31%) frame drops and submillisecond accuracy, our preliminary tests indicate that the newer version of the hardware (Raspberry Pi 3 with Raspberry Pi camera module v2) is capable of acquiring at 100 Hz at submillisecond temporal accuracy without dropping frames.

Correlated with the debate about the exact shape of theta phase precession, there is an ongoing debate about the exact shape of the place field. The shape of the place field is critical to the two competing models of theta phase precession: whereas the ramp excitation model requires asymmetric place field with a slow increase in firing rate as the rat approaches the location, with peak firing rate and a rapid fall-off in firing rate as the rat exits the place field (Mehta et al. 2002), the oscillatory interference model requires a symmetric place field (O’Keefe and Recce 1993; Burgess and O’Keefe 2011). Although the criteria used for defining the place field can affect the estimated asymmetry, precise position tracking and the consequent increased confidence in the exact shape of the place field can further help resolve this question.

In summary, the Picamera system described in the present work satisfies all the criteria desirable in an efficient tracking system for use with large and complex environments: easy to use, adaptable, temporally accurate, low cost, scalable, open source, and easily available. The system overcomes the bottleneck on tracking animal behavior in large spaces, therefore reducing the gap between the natural environments and experimental setup.

## GRANTS

This work was supported by Wellcome Trust/DBT India Alliance Grant IA/S/13/2/501024.

## DISCLOSURES

No conflicts of interest, financial or otherwise, are declared by the authors.

## AUTHOR CONTRIBUTIONS

S.S.D. conceived and designed research; R.S. and W.B. performed experiments; R.S. analyzed data; R.S. and S.S.D. interpreted results of experiments; R.S. prepared figures; R.S. and S.S.D. drafted manuscript; R.S., W.B., and S.S.D. edited and revised manuscript; R.S., W.B., and S.S.D. approved final version of manuscript.
